# In silico drug repositioning based on integrated drug targets and canonical correlation analysis

**DOI:** 10.1186/s12920-022-01203-1

**Published:** 2022-03-06

**Authors:** Hailin Chen, Zuping Zhang, Jingpu Zhang

**Affiliations:** 1 School of Software, East China Jiaotong University, Nanchang, 330013 China; 2 School of Computer Science and Engineering, Central South University, Changsha, 410083 China; 3 School of Computer and Data Science, Henan University of Urban Construction, Pingdingshan, 467000 China

**Keywords:** Drug repositioning, Canonical correlation analysis, Integrated targets

## Abstract

**Background:**

Besides binding to proteins, the most recent advances in pharmacogenomics indicate drugs can regulate the expression of non-coding RNAs (ncRNAs). The polypharmacological feature in drugs enables us to find new uses for existing drugs (namely drug repositioning). However, current computational methods for drug repositioning mainly consider proteins as drug targets. Meanwhile, these methods identify only statistical relationships between drugs and diseases. They provide little information about how drug-disease associations are formed at the molecular target level.

**Methods:**

Herein, we first comprehensively collect proteins and two categories of ncRNAs as drug targets from public databases to construct drug–target interactions. Experimentally confirmed drug-disease associations are downloaded from an established database. A canonical correlation analysis (CCA) based method is then applied to the two datasets to extract correlated sets of targets and diseases. The correlated sets are regarded as canonical components, and they are used to investigate drug’s mechanism of actions. We finally develop a strategy to predict novel drug-disease associations for drug repositioning by combining all the extracted correlated sets.

**Results:**

We receive 400 canonical components which correlate targets with diseases in our study. We select 4 components for analysis and find some top-ranking diseases in an extracted set might be treated by drugs interfacing with the top-ranking targets in the same set. Experimental results from 10-fold cross-validations show integrating different categories of target information results in better prediction performance than only using proteins or ncRNAs as targets. When compared with 3 state-of-the-art approaches, our method receives the highest AUC value 0.8576. We use our method to predict new indications for 789 drugs and confirm 24 predictions in the top 1 predictions.

**Conclusions:**

To the best of our knowledge, this is the first computational effort which combines both proteins and ncRNAs as drug targets for drug repositioning. Our study provides a biologically relevant interpretation regarding the forming of drug-disease associations, which is useful for guiding future biomedical tests.

**Supplementary Information:**

The online version contains supplementary material available at 10.1186/s12920-022-01203-1.

## Background

Over 100 years ago, the Nobel laureate Paul Ehrlich established his revolutionary ‘magic bullet’ concept, which has successfully inspired generations of chemists and pharmacologists to create target-specific drugs for disease treatment [1]. This declared paradigm has become a pragmatic criterion in drug discovery for the past decades. However, the interpretation of the magic bullet as a drug which acts through a single crucial target in an exclusive and highly specific way has been challenged, because increasing studies demonstrate drugs usually have multiple physiological targets rather than one target [2–4].

The polypharmacological feature in drugs enables us to find new indications (also known as drug repositioning [5]) for existing drugs. For instance, a study conducted by Skrott et al. [6] found that the metabolite of disulfiram binds to a new target NPL4, which is responsible for anti-cancer effects. Therefore, the old alcohol-aversion drug can be repurposed for tumour treatment. Meanwhile, unintended ‘off-targets’ may cause adverse drug reactions (ADR) [7], which would limit the use of drugs. It is therefore necessary to discover the real targets implicated in drug indications.

There are 4 potential types of macromolecules in biological systems with which we can interfere using small-molecule drugs: proteins, polysaccharides, lipids and nucleic acids [8]. Previous research efforts were mainly made on the first type of molecular targets [9–12]. The most recent studies in pharmacogenomics have discovered that drugs can regulate the expression levels of two categories of ncRNAs, namely miRNAs and lncRNAs. For example, Smith et al. [13] revealed that the expression levels of 44 miRNAs are repressed during glucocorticoid-induced apoptosis. Guo et al. [14] identified aspirin can activate the expression of a lncRNA named *OLA1P2* in human colorectal cancer. Given the intriguing fact that ncRNAs play significant roles in disease development [15–17], targeting these ncRNAs with small-molecule drugs offers another new and promising type of therapy for human diseases [18–23].

As traditional biomedical experiments are expensive and time-consuming, computational approaches provide an alternative tool for drug repositioning. For example, Chen et al. [24] exploited multiple heterogeneous data to integrate drug-disease network and drug–target network into one coherent model, and applied cross-network embedding to predict drug-disease associations for drug repositioning. A comprehensive and detailed survey on computational drug repositioning is available at Review [25]. Note that previous computational approaches for drug repositioning seldom take integrated target information into consideration. They usually exploit proteins as drug targets. We argue that integrating different types of targets would provide a better and more comprehensive understanding of drug’s MoA. Further, these methods discover only statistical associations between drugs and diseases at data level. They seldom investigate how drug-disease associations are formed at the molecular target level.

In this paper, we first comprehensively select drug targets from proteins, miRNAs and lncRNAs to construct drug–target interactions. Therapeutically verified drug indications are downloaded to form drug-disease associations. Then, we apply a CCA-based method to extract correlated sets of targets and diseases. The correlated targets and diseases provide explanations of the forming of drug-disease associations. We finally predict novel drug-disease associations for drug repositioning by combining the correlated sets. Comprehensive experiments demonstrate using integrated target information not only improves prediction performance, but also provides a more extensive view of drug’s MoA. Case studies suggest some top predictions are confirmed by existing databases. When compared with other methods using the benchmark datasets in our study, our approach shows improvements in terms of AUC value.

## Results

### Preliminary analysis of the datasets

In total, we receive 1190 drugs with both target and indication information. For the 1190 drugs, we obtain 5331 drug–target interactions containing 1668 targets and 5869 drug-disease associations including 1111 diseases. An overview of the two datasets is available at Tables 1 and 2, respectively.

**Table 1:** Statistics of the drug–target interactions used in our manuscript

Name	Statistics
# drugs	1190
# total targets (including proteins, miRNAs and lncRNAs)	1668
# proteins	1167
# miRNAs	348
# lncRNAs	153
# total drug–target interactions	5331
# drug–protein interactions	4337
# drug–miRNA interactions	825
# drug–lncRNA interactions	169
Average number of targets for each drug	4.5

**Table 2:** Statistics of the drug-disease associations used in our manuscript

Name	Statistics
# drugs	1190
# diseases	1111
# drug-disease associations	5869
Average number of associated diseases for each drug	4.9

We further use a boxplot (Additional file 1) to describe the distribution of numbers of targets and indications of the 1190 drugs. We discover that there are 885 (74.4%) drugs whose target numbers are less than 4.5 (the average value) and 887 (80.0%) drugs whose indication numbers are less than 4.9 (the average value). Meanwhile, as a category of newly discovered targets, the number of experimentally supported drug–ncRNA interactions are far less than that of drug–protein interactions. We can conclude from the analysis that our knowledge about drug–target interactions and drug-disease associations is not complete.

### Performance evaluation

In this study, we collect both proteins and ncRNAs as drug targets. We therefore separately use proteins, ncRNAs and integrated targets to conduct 10-fold cross-validation experiments. We use average AUC values for performance evaluation. The results are summarized in Table 3. We discover that integrating both proteins and ncRNAs results in better prediction performance than only using proteins or ncRNAs as targets. We also find that imposing sparsity constraint on CCA can improve prediction performance. Note almost all elements in the weight vectors in ordinary CCA (OCCA) are non-zero, indicating that OCCA cannot select a small number of features as informative drug targets and indications.

**Table 3:** Average AUC values received from the CCA methods based on 10-fold cross-validations

	SCCA (proteins + ncRNAs)	SCCA (ncRNAs)	SCCA (proteins)	OCCA (proteins + ncRNAs)	OCCA (ncRNAs)	OCCA (proteins)
AUC value	**0.8576**	0.7391	0.8537	0.8107	0.7283	0.8106

The bold value indicated the highest one

### Effects of parameters on cross-validation experiments

There are three parameters (*c*_1_, *c*_2_ and *k*) in our method. The parameters *c*_1_ and *c*_2_ are to control the sparsity. The parameter *k* is the number of canonical components. For simplicity, we choose the same value for *c*_1_ and *c*_2._ We comprehensively set the values of *c*_1_ and *c*_2_ in the range of [0.1, 0.9], and the value of *k* in the range of [60, 500] when conducting 10-fold cross validations. We list the average AUC values in Table 4. We find the best inference performance is achieved when *c*_1_ = *c*_2_ = 0.1, and *k* = 400.

**Table 4:** Average AUC values received based on 10-fold cross-validations by parameter tuning

	*k* = 60	80	100	200	300	400	500
*c*_1_ = *c*_2_ = 0.1	0.8146	0.8244	0.8293	0.8463	0.8542	**0.8576**	0.8575
0.3	0.8124	0.8124	0.8107	0.8027	0.8012	0.8014	0.8003
0.5	0.8146	0.8099	0.8026	0.7753	0.7717	0.7686	0.7649
0.7	0.8160	0.8107	0.8043	0.7752	0.7702	0.7659	0.7645
0.9	0.8160	0.8106	0.8042	0.7751	0.7702	0.7659	0.7645

The bold value indicated the highest one

### Investigating drug’s MoA at the molecular target level

Drugs exert their therapeutic effects through modulating their biological targets, and in turn promote healthy functioning of our metabolic system. As a drug usually has multiple targets, detecting the real target(s) implicated in a disease is critical for understanding drug’s MoA and for further drug repositioning.

We obtain 400 canonical components (Additional file 2) which correlate targets with diseases. We use four components (#1, #3, #6 and #7) as examples to investigate the biological meaning of the extracted sets of targets and diseases. We select the top targets and diseases in each component for analysis.

In component #1, there are 34 targets and 23 diseases with positive weight. We find from the database DisGeNET [26] that 4 high-ranking target proteins, *Interleukin-1 beta* (3rd), *Caspase-1* (3rd), *Caspase-3* (3rd) and *Matrix metalloproteinase-9* (3rd), are associated with the top disease *Periodontitis* (1st). Two top-scoring targets, *Interleukin-1 beta* (3rd) and *Matrix metalloproteinase-9* (3rd), are related with one top-scoring disease *Cholera* (4th). The target *Caspase-3* (3rd) is associated with the disease *Chlamydia trachomatis infection of genital structure* (5th).

Similar findings are discovered in component #3, #6 and #7. We list the confirmed top target-disease associations in the three components in Additional file 3, 4 and 5, respectively. Besides proteins, ncRNAs are found to be associated with diseases. For example, we discover in component #3 the top-ranking miRNA (*miR-135b*) is related with *malignant neoplasm of thyroid* (4th), *malignant neoplasm of lung* (6th) and *breast carcinoma* (7th), and the top-ranking miRNA (*miR-520h*) is associated with *malignant neoplasm of lung* (6th) and *breast carcinoma* (7th). These relationships are confirmed by the database HMDD [15]. In component #7, a lncRNA *UCA1* (8th) is found to be related with *Leukemia, Myeloid, Chronic-Phase* (3rd), which is verified by the database LncRNADisease [16]. Based on these findings, we presume drugs may act on the top-ranking targets in one canonical component to treat the top-ranking diseases in the same component.

### Comparison with other methods

As mentioned before, this is the first computational effort using integrated targets for drug repositioning. Previous computational approaches for drug repositioning were developed based on different data features they analysed. We therefore choose 3 other methods which can take our datasets as inputs for comparison. The 3 baseline methods are as follows:

- DBSI [27]: a collaborative-filtering-based method using chemical similarity for drug–target interaction prediction.
- SDTNBI [28]: an integrated tool for large-scale drug–target interaction prediction using chemical substructures.
- MLKNN [29]: a multi-label k-nearest neighbour method for drug side effect prediction.

To make fair comparison, we apply the 3 methods to our datasets and use 10-fold cross-validations for prediction performance comparison. For the method DBSI, we calculate drug–drug similarity according to Jaccard score based on their target information. This strategy of similarity calculation has been applied in other studies [30, 31]. The received AUC values for these methods are shown in Table 5. We perform Wilcoxon rank sum tests between SCCA and the other 3 methods based on the AUC values. The calculated *p* values are available at Table 6. The experimental results demonstrate our approach SCCA performs best in the 4 methods. Note that the other 3 methods cannot provide clues for biological interpretation.

**Table 5:** Comparison of average AUC values with existing methods based on 10-fold cross-validations

	SCCA	DBSI	SDTNBI	MLKNN
AUC value	**0.8576** ± 0.0005	0.8413 ± 0.0022	0.8395 ± 0.0010	0.7945 ± 0.0002

The bold value indicated the highest one

**Table 6:** The *p*-values received from Wilcoxon rank sum tests

	DBSI	SDTNBI	MLKNN
*p* value between SCCA and another method based on AUC values	1.6305E−04	1.7168E−04	1.6973E−04

### New indication prediction for existing drugs

After confirming the prediction ability, we further apply our method to those drugs, which are not in the benchmark datasets but whose target information is available, for their new indication predictions. There are 789 drugs of such kind. All known information, including drug–target interactions and drug-disease associations, in our gold-standard datasets is used for training. The potential indications are prioritized based on the prediction scores in descending order according to the method SCCA.

**Fig. 1:**
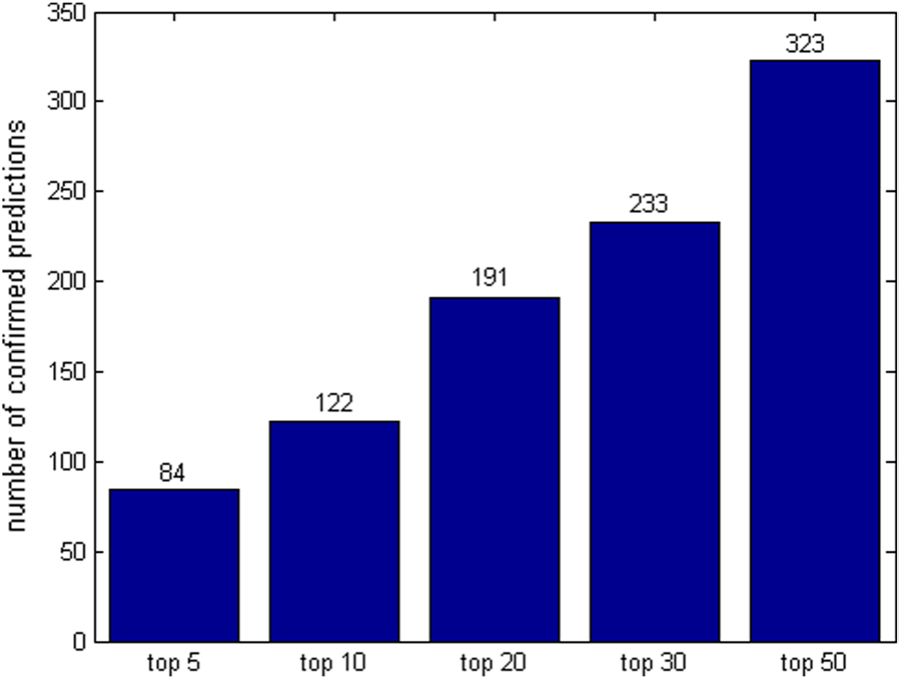
The numbers of validated indications by CTD in the top *k* predictions for the 789 drugs

We list the top 50 predicted results of the 789 drugs in Additional file 6 for future screenings. We further validate the top *k* (*k* = 5, 10, 20, 30 and 50) predictions by checking the public database CTD [32], a knowledgebase that houses information of chemicals, genes, phenotypes, diseases and exposures to advance understanding about human health. As this database contains both inferred and curated records, we only select curated drug-disease associations for prediction confirmation. The numbers of confirmed drug indications in the top *k* predictions are illustrated in Fig. 1. Because of space limitation, we only report the top 1 drug indication predictions supported by CTD in Table 7. More detailed information of the verified drug-disease associations in the top 50 predictions is available at Additional file 7. The excellent results indicate our method can be applied in real situations.

**Table 7:** The confirmed results in the top 1 drug indication predictions by CTD

Drug name	Disease name	Ranking in the prediction list	Evidence
Troglitazone	Hypertriglyceridemia	Top 1	CTD
Methysergide	Migraine disorders	Top 1	CTD
Ropivacaine	Pruritus	Top 1	CTD
Tenofovir disoproxil	HIV infections	Top 1	CTD
Remoxipride	Schizophrenia	Top 1	CTD
Rosiglitazone	Hypercholesterolemia	Top 1	CTD
Cerivastatin	Hypercholesterolemia	Top 1	CTD
Meperidine	Pain	Top 1	CTD
Dronabinol	Obesity	Top 1	CTD
Phenindione	Thromboembolism	Top 1	CTD
Amodiaquine	Malaria, falciparum	Top 1	CTD
Alfentanil	Pain	Top 1	CTD
Risedronic acid	Osteoporosis, postmenopausal	Top 1	CTD
Levobupivacaine	Pruritus	Top 1	CTD
Ketamine	Pain	Top 1	CTD
Sulfadoxine	Malaria, falciparum	Top 1	CTD
Methotrimeprazine	Schizophrenia	Top 1	CTD
Acenocoumarol	Thromboembolism	Top 1	CTD
Diamorphine	Pain	Top 1	CTD
Pimavanserin	Schizophrenia	Top 1	CTD
Ciprofibrate	Hypertriglyceridemia	Top 1	CTD
Vitamin d	Hypoparathyroidism	Top 1	CTD
Elagolix	Endometriosis	Top 1	CTD
mg132	Multiple myeloma	Top 1	CTD

## Discussion

Uncovering drug’s MoA is of great importance for drug repositioning. In vivo and in vitro experiments are useful but expensive tools to address the problem. Our CCA-based computational method provides an alternative to revealing the targets which are implicated in drug indications, and results suggest the extracted sets of targets and diseases are biologically meaningful. Compared with previous studies, we integrate both proteins and ncRNAs as drug targets. Experiments further demonstrate using integrated targets improves prediction performance.

Even though, our proposed method has been shown to be useful in drug repositioning. Some limitations in this study need to be pointed out. First, our method depends heavily on known drug–target interactions and drug-disease associations. As we know, many drug targets (especially drug–ncRNA interactions) and drug indications have not been discovered. The incompleteness of data would result in biased prediction results. We expect combining more experimentally confirmed drug–target interactions and drug-disease associations would provide more reliable predictions. Meanwhile, there are 3 parameters in our method. Selecting appropriate values for the 3 parameters to receive optimal results is a challenging task. Third, the numbers of extracted components are determined by the parameter *k* (see Eq. 4) in our method, and different numbers of extracted components would influence our interpretation of drug’s MoA.

More recently, a growing number of studies [24, 33–43]are exploiting both features from drugs and diseases for drug repositioning. Integrating these features may provide more reliable prediction results. Another trend in drug repositioning is drug combinations [44–46] (see Review [47] for more details), which can result in low adverse side effects and high treatment efficacy compared to single drug administration. We believe these efforts offer help with drug discovery and disease treatment from different perspectives.

## Conclusions

In this study, we apply a CCA-based method to extract correlated sets of targets and diseases, and the correlated targets and diseases provide clues for explaining drug’s MoA for drug repositioning. We further propose a prediction scheme for drug repositioning based on the extracted correlated sets. Experimental results of cross-validations indicate that integrating different categories of targets and imposing sparsity constraint on CCA improve prediction performance. Case studies demonstrate that some of the top predictions by our method are supported by literature. Moreover, our method shows improvement in prediction accuracy when compared with other approaches. We expect that our study offers a useful tool for drug repositioning.

## Methods

### Data preparation

We collect two datasets, namely drug–target interactions and drug-disease associations, from public databases for our study. The two datasets are regarded as gold-standard data. We use the benchmark datasets to evaluate the performance of our method. We also use the two datasets as training datasets for comprehensive indication prediction.

For drug–target interactions, we integrate 3 categories of macromolecules (proteins, miRNAs and lncRNAs) as drug targets. We obtain drug–protein interactions from DrugBank [48], a freely available web resource containing detailed information about drugs, their mechanisms, their interactions and their targets. We only select small molecule drugs and approved targets in DrugBank in our study. We download drug–miRNA interactions and drug–lncRNA interactions from SM2miR [49] and D-lnc [50], respectively. The two databases separately provide comprehensive repositories to detect the modification of drugs on miRNA and lncRNA expression. We restrict the species to *Homo sapiens* in both databases. We do not take inferred results in D-lnc for consideration.

Drug-disease associations are received from repoDB [51], a database consisting of approved and failed drugs and their indications. We only keep the approved drug-indication pairs in the database in our datasets.

### Method description

Suppose that we have a set of *m* drugs with *p* molecular target features and *q* disease features. We denote each drug by a target feature vector t = (t_1_, t_2_, t_3_, … t_*p*_)^*T*^ and by a disease feature vector d = (d_1_, d_2_, d_3_, … d_*q*_)^*T*^, where t_*i*_ (or d_*j*_) is represented for the presence or absence of a target (or a disease) by 1 or 0, respectively.

Consider two linear combinations for targets and diseases as $u_{i} = \alpha^{T} t_{i}$ and $v_{i} = \beta^{T} d_{i}$(*i* = 1, 2, 3, …, *m*), where *α* = (*α*_1_, *α*_2_, *α*_3_, … *α*_*p*_)^*T*^ and *β* = (*β*_1_, *β*_2_, *β*_3_, … *β*_*q*_)^*T*^are weight vectors. We apply canonical correlation analysis [52] to find weight vectors *α* and *β* which maximize the following correlation coefficient:

1 $$\rho = corr(u,v) = \frac{{\sum\nolimits_{i = 1}^{m} {\alpha^{T} t_{i} \cdot \beta^{T} d_{i} } }}{{\sqrt {\sum\nolimits_{i = 1}^{m} {\left( {\alpha^{T} t_{i} } \right)^{2} } } \sqrt {\sum\nolimits_{i = 1}^{m} {\left( {\beta^{T} d_{i} } \right)^{2} } } }}$$

Let *X* denote an *m* × *p* matrix and *Y* denote an *m* × *q* matrix. Then the maximization problem can be formally rewritten as follows:

2 $$\max imize\{ \alpha^{T} X^{T} Y\beta \} \;{\text{subject to}}\;\left\| \alpha \right\|_{2}^{2} \le 1\,\left\| \beta \right\|_{2}^{2} \le 1.$$

We refer to it as ordinary canonical correlation analysis (OCCA). OCCA usually results in vectors *α* and *β* with many non-zero elements. To impose sparsity on *α* and *β*, we choose to add penalties to (2) like reference [53–55] and the maximization problem is considered as:

3 $$\begin{aligned} & \max imize\left\{ {\alpha^{T} X^{T} Y\beta } \right\} \\ & \quad {\text{subject to}}\;\left\| \alpha \right\|_{2}^{2} \le 1,\;\left\| \beta \right\|_{2}^{2} \le 1,\;\left\| \alpha \right\|_{1} \le c_{1} \sqrt p \;\left\| \beta \right\|_{1} \le c_{2} \sqrt q \\ \end{aligned}$$

where *c*_1_ and *c*_2_ are parameters to control the sparsity. We refer to this as sparse canonical correlation analysis (SCCA). We apply a strategy of penalized matrix decomposition (PMD) [56] to the matrix $Z{ = }X^{T} Y$ to obtain the weight vectors *α* and *β*.

To receive multiple canonical variates, we use a deflation manipulation iteratively as follows:

4 $$Z^{k + 1} = Z^{k} - d_{k} \alpha_{k} \beta_{k}^{T}$$

where $\alpha_{k}$ and $\beta_{{_{k} }}^{{}}$ are the weight vectors, and *d*_*k*_ is the singular value obtained in each iteration step. We choose targets and diseases in the *k* pairs of weight vectors with the highest values as correlated sets.

To predict new indications for a drug with a known target vector *x*_*new*_, we compute the scores of *y*_*new*_ by combining the *k* pairs of weight vectors according to the following equation:

5 $$y_{new} = \sum\limits_{i = 1}^{k} {\beta_{i} \rho_{i} \alpha_{i}^{T} x_{new} }$$

**Fig. 2:**
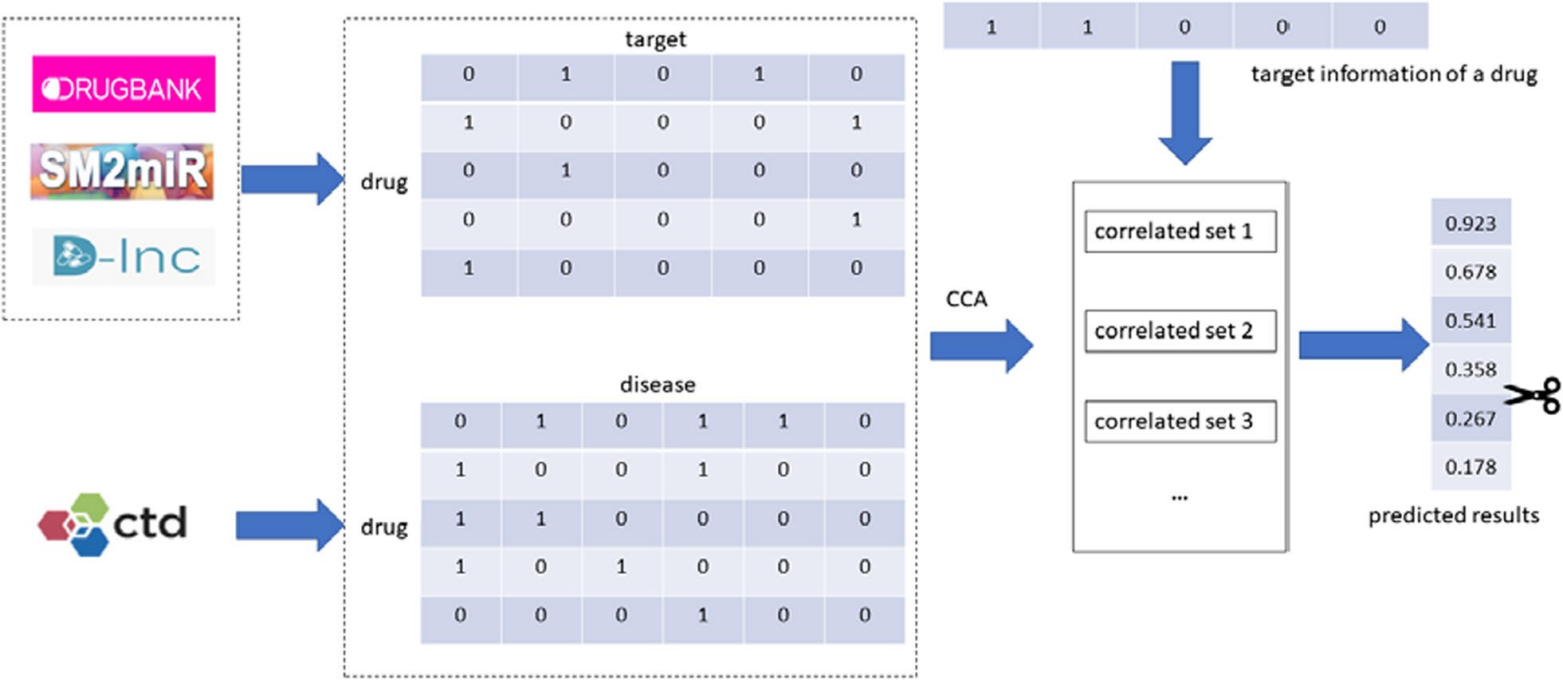
The workflow of our proposed method. Drug–target interactions and drug-disease associations are first downloaded from public databases. CCA is then applied to the two datasets to extract correlated sets. Finally, new drug-disease associations are predicted by combining the extracted sets. The top predictions are selected as new indications for drugs of interest.

The elements in *y*_*new*_ with the highest scores are chosen as the predicted indications for the drug. This prediction strategy was used in previous studies [53, 54]. The workflow of our method is depicted in Fig. 2.

### Evaluation metrics

In order to test the prediction performance of our method, we implement 10-fold cross-validations on the drugs. We split the whole drugs into 10 subsets of roughly equal sizes, and each subset is used in turn as a test set. We train our method on the remaining 9 subsets. We prioritize the inferred drug-disease associations according to the prediction scores (see Eq. (5)). Setting different thresholds, true positive rate (TPR) and false positive rate (FPR) are calculated to plot ROC curves. Area under ROC curve (AUC) values are computed for performance evaluation. To obtain robust results, we repeated the cross-validation experiments 10 times.

Moreover, we comprehensively predict novel drug-disease associations for drug repositioning for the drugs not included in the benchmark datasets. We analyse the top-ranked results by searching evidence from the public database CTD [32]. Note we only choose curated records of drug indications in this database for prediction confirmation.

## Supplementary Information

**Additional file 1**. Distribution of numbers of targets and indications of the 1190 drugs.

**Additional file 2**. The extracted 400 correlated sets by SCCA.

**Additional file 3**. Confirmed top-ranking target-disease associations in component #3.

**Additional file 4**. Confirmed top-ranking target-disease associations in component #6.

**Additional file 5**. Confirmed top-ranking target-disease associations in component #7.

**Additional file 6**. The top 50 predicted indications for the 789 drugs.

**Additional file 7**. The verified drug-disease associations in the top 50 predictions.

**Additional file 8**. The source code and data sets used in this study.

## Data Availability

All data used in this study are available from the DrugBank/SM2miR/D-lnc/repoDB /CTD/DisGeNET/HMDD/LncRNADisease databases. The original publications of these databases have been cited in our manuscript. The links for these databases are as follows. The source code and data sets used in this study are available at Additional file 8. DrugBank: https://go.drugbank.com/. SM2miR: http://www.jianglab.cn/SM2miR/. D-lnc: http://www.jianglab.cn/D-lnc/. repoDB: http://apps.chiragjpgroup.org/repoDB. CTD: http://ctdbase.org/. DisGeNET: http://www.disgenet.org. HMDD: http://www.cuilab.cn/hmdd. LncRNADisease: http://www.cuilab.cn/lncrnadisease

## Abbreviations

ncRNA: Non-coding RNAs
CCA: Canonical correlation analysis
MoA: Mechanism of actions
ADR: Adverse drug reactions
PMD: Penalized matrix decomposition
