# Supplementary figures and images for: In silico drug repositioning based on integrated drug targets and canonical correlation analysis
Source: BMC Med Genomics. 2022 Mar 6;15:48. doi: 10.1186/s12920-022-01203-1 (PMC8898485; doi:10.1186/s12920-022-01203-1)

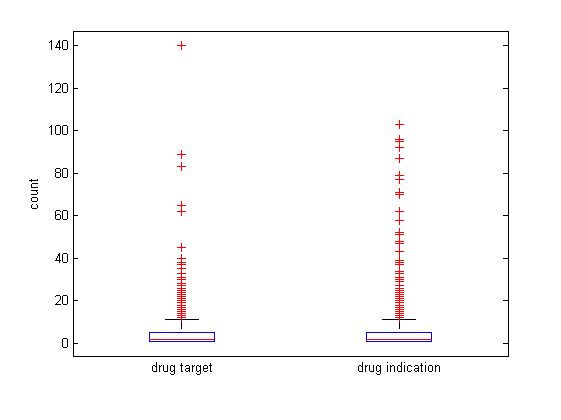
 **Additional file 1** Distribution of numbers of targets and indications of the 1190 drugs

Supplement: Supplementary file 1 — Additional file 1. Distribution of numbers of targets and indications of the 1190 drugs. [file 12920_2022_1203_MOESM1_ESM.docx]
