# Supplementary material for: In silico drug repositioning based on integrated drug targets and canonical correlation analysis
Source: BMC Med Genomics. 2022 Mar 6;15:48. doi: 10.1186/s12920-022-01203-1 (PMC8898485; doi:10.1186/s12920-022-01203-1)
Supplement: Supplementary file 3 — Additional file 3. Confirmed top-ranking target-disease associations in component #3. [file 12920_2022_1203_MOESM3_ESM.docx]

**Additional file 3**– Confirmed top-ranking target-disease associations in component #3

| target name | target type | ranking in the target list | disease name | ranking in the disease list | evidence |
| --- | --- | --- | --- | --- | --- |
| Nucleolar and coiled-body phosphoprotein 1 | protein | 1 | Small cell carcinoma of lung | 3 | DisGeNET |
| Nucleolar and coiled-body phosphoprotein 1 | protein | 1 | Malignant neoplasm of lung | 6 | DisGeNET |
| Nucleolar and coiled-body phosphoprotein 1 | protein | 1 | Breast Carcinoma | 7 | DisGeNET |
| miR-135b | miRNA | 1 | Malignant neoplasm of thyroid | 4 | HMDD |
| miR-135b | miRNA | 1 | Malignant neoplasm of lung | 6 | HMDD |
| miR-135b | miRNA | 1 | Breast Carcinoma | 7 | HMDD |
| miR-520h | miRNA | 1 | Malignant neoplasm of lung | 6 | HMDD |
| miR-520h | miRNA | 1 | Breast Carcinoma | 7 | HMDD |
