# Supplementary material for: In silico drug repositioning based on integrated drug targets and canonical correlation analysis
Source: BMC Med Genomics. 2022 Mar 6;15:48. doi: 10.1186/s12920-022-01203-1 (PMC8898485; doi:10.1186/s12920-022-01203-1)
Supplement: Supplementary file 4 — Additional file 4. Confirmed top-ranking target-disease associations in component #6. [file 12920_2022_1203_MOESM4_ESM.docx]

**Additional file 4**– Confirmed top-ranking target-disease associations in component #6

| target name | target type | ranking in the target list | disease name | ranking in the disease list | evidence |
| --- | --- | --- | --- | --- | --- |
| 3-hydroxy-3-methylglutaryl-coenzyme A reductase | protein | 1 | Arteriosclerosis | 2 | DisGeNET |
| 3-hydroxy-3-methylglutaryl-coenzyme A reductase | protein | 1 | Hyperlipidemia, Familial Combined | 3 | DisGeNET |
| 3-hydroxy-3-methylglutaryl-coenzyme A reductase | protein | 1 | Hypertriglyceridemia | 7 | DisGeNET |
| 3-hydroxy-3-methylglutaryl-coenzyme A reductase | protein | 1 | Hypercholesterolemia | 8 | DisGeNET |
| Integrin alpha-L | protein | 2 | Arteriosclerosis | 2 | DisGeNET |
| Integrin alpha-L | protein | 2 | Hypercholesterolemia | 8 | DisGeNET |
