# Supplementary material for: In silico drug repositioning based on integrated drug targets and canonical correlation analysis
Source: BMC Med Genomics. 2022 Mar 6;15:48. doi: 10.1186/s12920-022-01203-1 (PMC8898485; doi:10.1186/s12920-022-01203-1)
Supplement: Supplementary file 5 — Additional file 5. Confirmed top-ranking target-disease associations in component #7. [file 12920_2022_1203_MOESM5_ESM.docx]

**Additional file 5**– Confirmed top-ranking target-disease associations in component #7

| target name | target type | ranking in the target list | disease name | ranking in the disease list | evidence |
| --- | --- | --- | --- | --- | --- |
| Epithelial discoidin domain-containing receptor 1 | protein | 1 | Philadelphia chromosome-positive acute lymphoblastic leukemia | 1 | DisGeNET |
| Epithelial discoidin domain-containing receptor 1 | protein | 1 | Renal Cell Carcinoma | 9 | DisGeNET |
| Tyrosine-protein kinase ABL1 | protein | 3 | Philadelphia chromosome-positive acute lymphoblastic leukemia | 1 | DisGeNET |
| Tyrosine-protein kinase ABL1 | protein | 3 | Leukemia, Myeloid, Chronic-Phase | 3 | DisGeNET |
| Tyrosine-protein kinase ABL1 | protein | 3 | Renal Cell Carcinoma | 9 | DisGeNET |
| UCA1 | lncRNA | 8 | Leukemia, Myeloid, Chronic-Phase | 3 | LncRNADisease |
